# Effects of acupuncture at limb Acupoints-Guangming (GB37) on UDVA, CS, and EEG microstate in myopia

**DOI:** 10.3389/fnins.2024.1492529

**Published:** 2024-11-21

**Authors:** Zhongqing Wang, Hao Yan, Kangna Su, Ruixin Wu, Lihan Wang, Hongsheng Bi, Jianfeng Wu

**Affiliations:** ^1^Medical College of Optometry and Ophthalmology, Shandong University of Traditional Chinese Medicine, Jinan, China; ^2^Jinan High-tech East District Hospital, Jinan, China; ^3^Shanxi Eye Hospital, Xi’an People’s Hospital (Xi’an Fourth Hospital), Affiliated People’s Hospital of Northwest University, Xi’an, China; ^4^Affiliated Eye Hospital of Shandong University of Traditional Chinese Medicine, Shandong Institute of Eye Disease Prevention and Treatment, Shandong Provincial Key Laboratory of Integrative Medicine for Eye Diseases, Shandong Provincial Clinical Research Center of Ophthalmology and Children Visual Impairment Prevention and Control, Shandong Engineering Technology Research Center of Visual Intelligence, Shandong Institute of Children Health and Myopia Prevention and Control, Jinan, China

**Keywords:** acupuncture, uncorrected distance visual acuity, contrast sensitivity, microstates, EEG

## Abstract

**Introduction:**

Acupuncture is beneficial in improving visual function for myopi periocular acupoints Taiyang can improve contrast sensitivity (CS). In this study, we aim to further investigate the impact of acupuncture at the limbs acupoints-Guangming(GB37) acupoint on visual function, and the neural mechanism of acupuncture at the GB37 acupoint improving visual function through electroencephalography (EEG) microstate.

**Methods:**

A total of 22 myopia were recruited. Uncorrected distance visual acuity (UDVA) and CS of myopic patients were tested before and after acupuncture, and EEG data were recorded throughout the entire acupuncture procedure.

**Results:**

Our study found that compared with pre-acupuncture, the UDVA and CS of both eyes at each spatial frequency were improved; compared with the resting state of pre-acupuncture, the duration, occurrence and contribution of microstate A were significantly increased, while those of microstate D were decreased during the post-acupuncture state. The duration of microstate A was positively correlated with the CS. There was no correlation between UDVA and EEG microstates.

**Discussion:**

Acupuncture at GB37 can improve the UDVA and CS in myopic patients, which may be related to microstate A.

## Introduction

Myopia, being the most frequent refractive error, is a major contributor to reversible visual impairment and blindness across the globe (Han et al., 2022). Acupuncture as a supplementary treatment for myopia, is an affordably priced therapeutic approach that is slightly invasive. A previous study has shown that acupuncture is a favorable treatment on improving the adolescent UDVA (Tao et al., 2014). Research has illustrated that acupuncture could improve the best corrected visual acuity and increase mean light sensitivity of patients with nonarteritic anterior ischemic optic neuropathy (Qin et al., 2015). A recent study indicates that after acupuncture treatment at periocular acupoints and limbs acupoints such as Guangming et al., improvements are observed in UDVA and CS (Blechschmidt et al., 2017). Another study discovered that CS and visual function of patients with retinitis pigmentosa were significantly improved after acupuncture at periocular acupoints and limbs acupoints (Bittner et al., 2014). Fereydouni et al. found acupuncture at limbs acupoints such as *Guangming* et al. could improve UDVA in patients with retinitis pigmentosa (Fereydouni et al., 2017). A latest study indicates that acupuncture at Guangming et al. periocular acupoints and limbs acupoints can effectively enhance UDVA, refractive error, and stereopsis in children with anisometropic amblyopia (Ma et al., 2024). Previous studies have primarily concentrated on the effects of combined acupoints of periocular acupoints and limbs acupoints on visual function. In our previous work, we have investigated the needling periocular acupoint can improve CS in myopic patients. Furthermore, we have conducted preliminary exploration on the relationship between needling periocular acupoint and brain function (Su et al., 2023). In this study, we further investigate the effects of a single limbs acupoint-GB37 (a point frequently mentioned in previous literature) on the visual function of myopia and its potential neural mechanisms.

Acupuncture can activate sensory nerves and modulate the central nervous system (Si et al., 2021). Therefore acupuncture at specific points can improve the visual function of myopic patients by clearing meridians (Huang and Wu, 2016). The GB37 point, an acupoint of the gallbladder channel, is characterized as an effective acupoint that impacts a variety of vision-related disorders (Gareus et al., 2002). Modern scholars have confirmed through clinical and experimental research that the symptom of eye itch has been effectively added to the indications for using the acupuncture point GB37 (Huang and Wu, 2016). Research has shown that stimulating the GB37 acupuncture point can alleviate discomfort associated with eye strain (Huang, 2015). A recent Randomized-Controlled Trial found laser acupuncture GB37 could improve the symptoms of dry eye disease (Hu et al., 2021). Another study found acupuncture at GB37 could enhance visual acuity and optic nerve function in children with anisometropic amblyopia, potentially due to its regulatory effects on brain activities in the anterior cerebellum (Wang et al., 2024). Based on the significant role of the GB37 point in treating eye conditions, our study has selected it as the primary acupuncture point for research. The neural response in the vision cortex after acupuncture at GB37 persisted beyond the expected duration, and the functional magnetic resonance imaging (fMRI) research showed GB37 point can induce sophisticated brain activity in the visual cortex (Liu et al., 2013).

Contrast sensitivity refers to the ability to identify the difference between different brightness or color, which is an important index to evaluate visual function. Visual acuity measures visual performance specifically in high-contrast situations (Arikan and Kamis, 2022). In contrast, CS is able to assess visual capabilities under both low- and medium-contrast conditions, which plays a crucial role in everyday activities, including night driving, newspaper reading, and identifying object boundaries, and so on (Roark and Stringham, 2019; Nti et al., 2022). Zhang et al. discovered a significant positive correlation between the Amplitude of Low-Frequency Fluctuations (ALFF) and Regional Homogeneity (ReHo) in the right orbital part of the middle frontal gyrus and the best-corrected visual acuity of the right eye (Zhang et al., 2023). A study found CS was closely related to V1 cortex activity, and CS showed a positive correlation with both the overall size of V1 and localized cortical magnification, with a stronger correlation observed between greater cortical magnification and higher CS at the horizontal meridian compared to the vertical meridian (Himmelberg et al., 2022). In another study, CS was notably reduced particularly at lower spatial frequencies correlating with reductions in brain area V1 responses (Niemeyer and Paradiso, 2017). This suggests that methods like acupuncture used to boost CS in myopic patients might affect extensive brain regions or networks involved in brain function. The mechanism by which acupuncture on limb acupoints improves CS is unclear. Acupuncture could activate afferent nerves and modulate the central nervous system (Li C. et al., 2023). Li et al. believed that the mechanism of acupuncture’s effect might be linked to the activation and the interconnectedness of specific brain regions such as middle frontal gyrus, supramarginal gyrus, and inferior frontal gyrus (Li B. et al., 2022). Examining the influence of acupuncture on brain functions can help us to understand the central neural pathways involved in the acupuncture treatment of myopia.

To reveal the neural mechanisms underlying the acupuncture, EEG and fMRI were utilized in previous studies (Si et al., 2021; Xingjie et al., 2023). While EEG microstate analysis is traditionally used in cognitive and neural studies, recent research has shown its applicability in visual research as well, particularly in understanding brain network dynamics during visual tasks (Wang et al., 2023). EEG is a non-invasive technique that captures the brain’s spontaneous electrical activity with high temporal resolution. Nevertheless, fMRI studies have been unable to capture the temporal dynamics changes during acupuncture due to the technique’s poor temporal resolution (Bullmore and Sporns, 2009). Acupuncture serves as a method for modulating neural activity within the brain. Presently, an increasing number of studies are directing their attention toward examining the response of various network nodes and brain regions using fMRI (Chae et al., 2013; Ibrahim et al., 2021). However, the temporal dynamics of the brain during acupuncture treatment for myopia remain unclear. Exploring brain dynamics can be achieved through various neuroimaging techniques, each with its own temporal resolution. While high-resolution fMRI captures slower neural dynamics over seconds, EEG microstate analysis offers a faster millisecond-scale exploration of neural dynamic changes (Michel and Koenig, 2018; Schumacher et al., 2019). Earlier research demonstrated that EEG activity’s dynamic patterns can be empirically categorized into a sequence of quasi-stable topographic EEG maps, known as “EEG microstates” (Michel and Koenig, 2018). EEG microstate is a state in which the scalp potential field remains relatively stable during a specific period of time (Blechschmidt et al., 2017), which maintains its stability for a brief duration of 80–120 ms before swiftly changing to a different microstate (Michel and Koenig, 2018). Research has demonstrated the existence of four distinct EEG microstates (A, B, C, and D) are consistently reproducible among different participants (Koenig et al., 2002). And these dynamics are associated with four brain networks: the auditory network, visual network, salience network, and dorsal attention network (Britz et al., 2010). According Milz et al. study, microstate A is distinctly associated with visual processing, while microstate B is linked with verbal processing (Milz et al., 2016). It has been established that characteristics of the EEG microstate time series, such as duration, occurrence and transition probability (TP), are effective indicators for identifying various cognitive states in participants (Si et al., 2021). Acupuncture substantially influenced functional brain activity, facilitating communication between distinct areas of the brain (Yu et al., 2018). A previous study found acupuncture at limb point could increase the occurrence, duration, and contribution parameters of microstate C, while decreasing those of microstate D (Si et al., 2021). Our preliminary study has found that acupuncture at the eye-surrounding *Taiyang* point, the CS of both eyes in myopic patients at each spatial frequency was significantly improved and the contribution of microstate C was decreased, which was negative correlation with CS, while the transition probability from microstate A to microstate C was reduced (Su et al., 2023). GB37, as mentioned above, also has beneficial effects on vision. Though they target similar clinical outcomes, such as improving vision, they do not belong to the same meridian or collateral channels. *Taiyang* is an extra-meridian point, generally used for more localized conditions around the eyes, while GB37, being part of the Gallbladder meridian, may influence broader systemic pathways connected to the eyes.

However, it remains unclear whether acupuncture on GB37 acupoint can improve UDVA and CS of patients with myopia and the neural mechanism. In this study, we investigate the effects of acupuncture at the GB37 on the UDVA and CS in myopic patients, as well as the correlation between EEG microstates and UDVA and CS, further explaining the neural mechanism by which acupuncture on limb acupoint improves visual function.

## Materials and methods

### Subjects

A total of 22 myopia from Affiliated Eye Hospital of Shandong University of Traditional Chinese Medicine were enrolled in this study, including 10 males and 12 females, aged 19–26 years (mean ± standard deviation: 22.90 ± 2.06 years). The average spherical equivalent power was −3.17 ± 1.06D. All subjects underwent left and right eye visual acuity examination separately. This study complies with the Declaration of Helsinki and is approved by the Institutional Ethics Committee of Clinical Trials of Eye Hospital Affiliated to Shandong University of Traditional Chinese Medicine (registration No. SDUTCM2024 0301102). Inclusion criteria as followed: Myopia ① were ≥ 1.0 the best corrected visual acuity of both eyes; ② were a spherical equivalent (SE) ≤ −0.50D, and > −6.00D (SE = spherical equivalent + 1/2 cylindrical diopter); ③ were cylinder power ≤ 1.50D and binocular anisometropia ≤1.00D; ④ were able to wear soft contact lenses to correct binocular vision, and the best-corrected visual acuity of both left and right eyes was ≥1.0; ⑤ had no other pathologies affecting visual acuity in both eyes; ⑥ were aged 19–26 years old; and ⑦ were right-handed. Exclusion criteria as followed: Myopia ① were unable to wear soft contact lenses; ② suffered from keratitis, conjunctivitis, glaucoma, nystagmus, eyelid fissure, or other concurrent organic ocular pathologies after ophthalmological examination; ③ had other systemic diseases or mental disorders, such as hypertension, claustrophobia, and neurodegenerative diseases; ④ were participating in other clinical trials; and ⑤ received acupuncture treatment in the past 3 months.

#### Visual function examination

The UDVA and CS of myopia were examined before and after acupuncture (20 min), and the order of left and right eye examination was randomized.

#### UDVA examination

Uncorrected distance visual acuity was checked with a standard logarithmic visual acuity chart (LED type 2.5 m, Hualong Medical Devices Co., Ltd.), and then converted to logMAR visual acuity for statistical analysis.

#### CS examination

The CS test was conducted using the visual function testing device (OPTEC6500, Stereo Optical Co., Inc., Chicago, IL, United States). The subject sat in a dark environment, with spatial frequencies set to 1.5, 3, 6, 12, and 18 cycles per degree (cpd), and the testing distance maintained at 6 meters. Without glare mode, choosing photopic conditions (85 cd/m^2^), the subject should be informed that the test grating includes vertical, leftward, and rightward directions. They are encouraged to read out the clearest direction visible at the end of the test. The final recognized grating was recorded as the CS value of that spatial frequency.

### Acupuncture experiment paradigm

**Figure 1:**
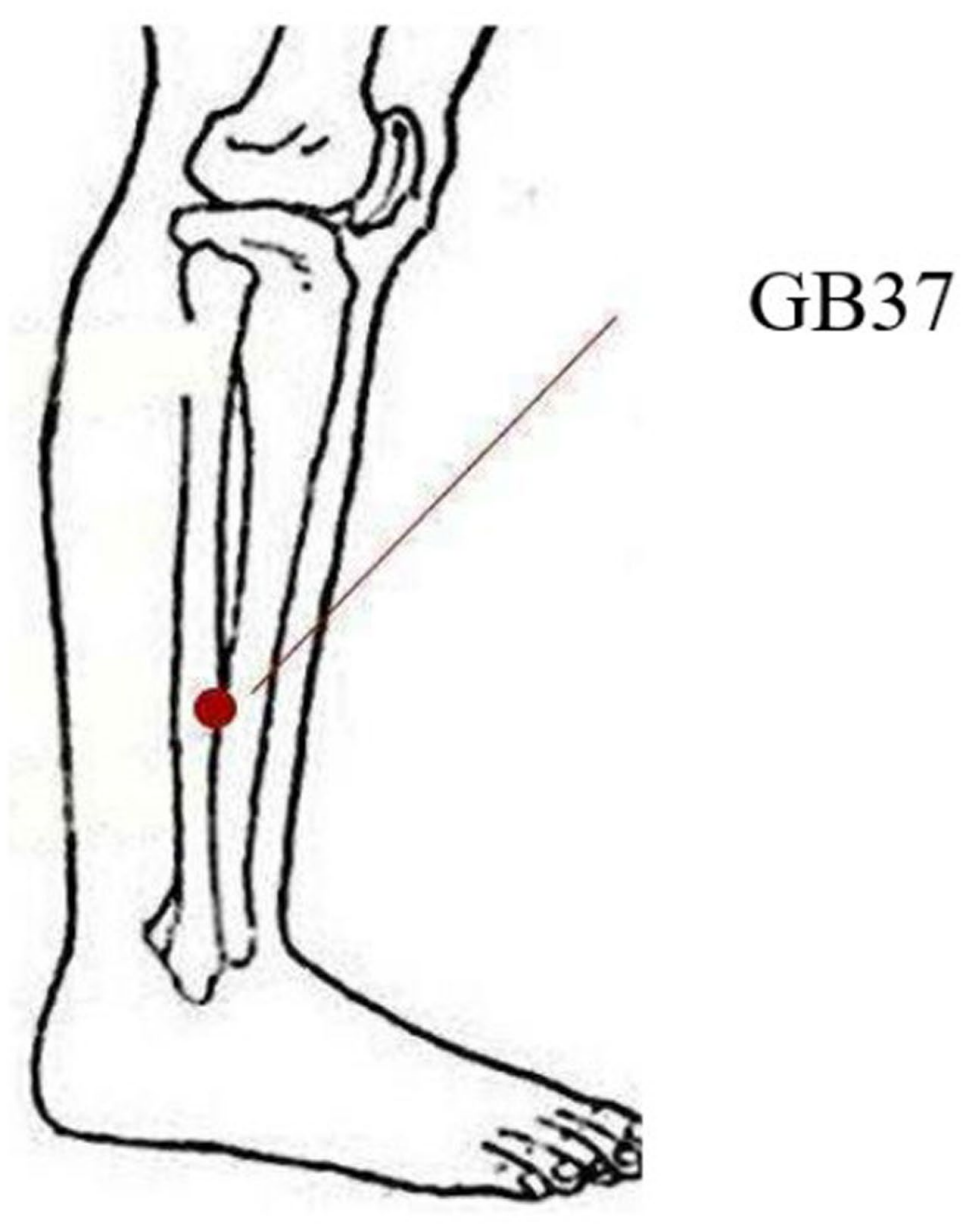
The location of the Guangming acupoint.

**Figure 2:**
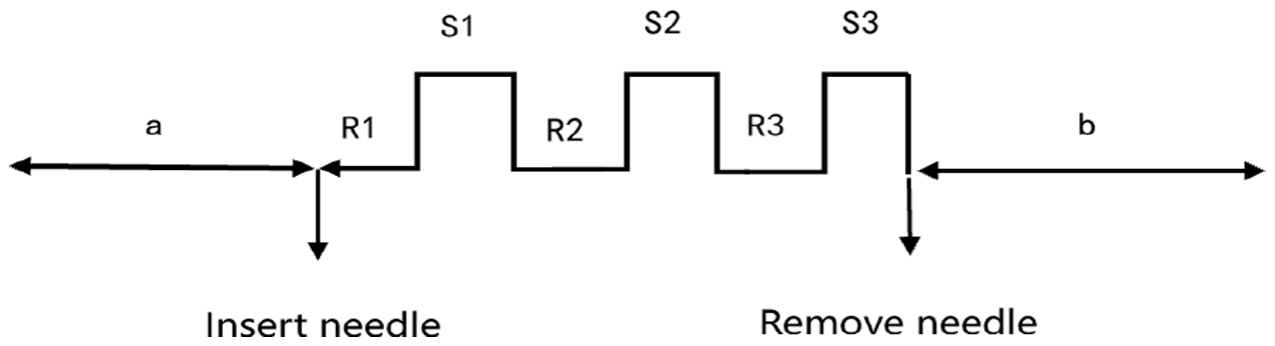
The process of needling the acupoint Guangming (GB37). a and b indicate 6 min the resting state of pre-acupuncture and the post acupuncture state, respectively; R1–R3 represents the 1-min needle retention state, and S1–S3 represents the 1 min needle running state.

Acupuncture on the GB37 point of the right lower limb was administered to myopic patients by the same acupuncturists to ensure that the results were not confounded by bilateral differences. The right side have been chosen in order to maintain consistency with our prior studies that focus on one side for particular therapeutic effects (Su et al., 2023). GB37 is located on the lateral side of the lower leg, 5 cun above the tip of the lateral malleolus, along the anterior edge of the fibula (Figure 1). During acupuncture, disposable sterile acupuncture needles (specification: 0.35 mm × 50 mm, Suzhou Huatuo Medical Co., Ltd.) were used. The acupuncture method was to retain the needle for 1 min after entering the deqi, followed by flattening reinforcing and reducing by twirling for 1 min with a twining frequency of about 100 times/min, and then removing the needle after repeating the operation for three times. Resting-state EEG signals from myopia was conducted for 6 min prior to acupuncture treatment and for another 6 min after removing the needle (Figure 2) (Su et al., 2023).

### EEG data collection and analysis

#### EEG recording

**Figure 3:**
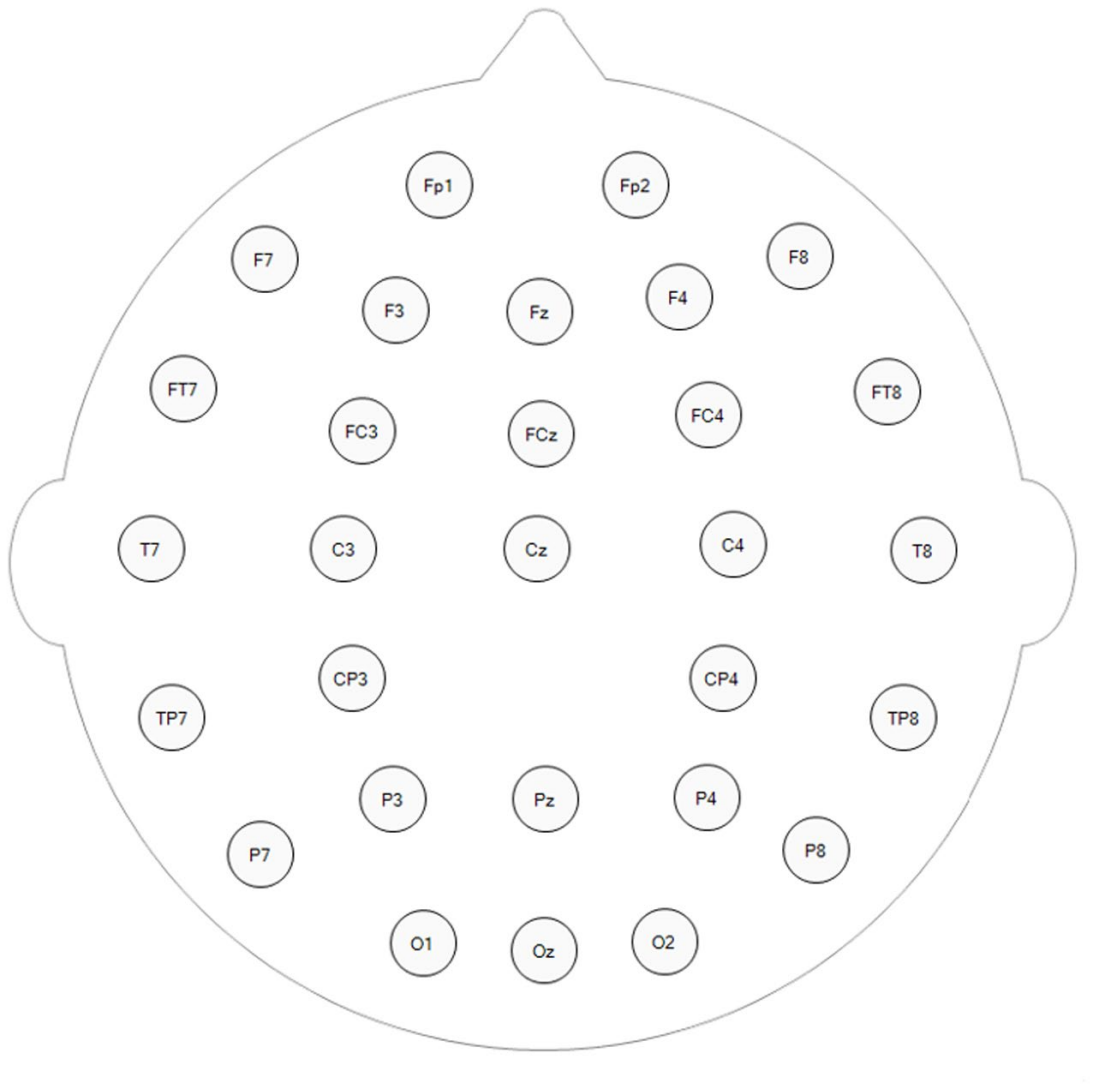
Electroencephalography (EEG) electrode positions on the brain.

Electroencephalography signals were recorded by a NicoletOne (Nicolet Company, United States) with 29 channels in the international 10–20 system (Figure 3), with Cpz serving as the reference electrode. The EEG signal was sampled at a frequency of 1,024 Hz, and EEG data were recorded during the entire acupuncture process (Figure 2). The study was carried out and managed utilizing E-prime Software (version 2.0, Psychological Software Tools, Pittsburgh, PA, United States), which managed the operational aspects and regulated the commencement and conclusion of the experiment.

#### EEG data preprocessing

Each patient’s EEG data was extracted for 6 min from the resting state and post-acupuncture state, respectively, and the EEG signal preprocessing was performed by the EEGLAB toolbox in MATLAB software (MathWorks Inc., Natick MA, United States). Then, a finite impulse response (FIR) filter was applied to the segmented data within the 0.5–60 Hz range for bandpass filtering, and a 50 Hz notch filter was applied to remove powerline noise (Pedroni et al., 2017). The artifact removal using AAR 1.3 plugin in EEGLAB was utilized for artifact removal, employing blind source separation (BSS) to eliminate electromyography and electrooculogram artifacts (Pascual-Marqui et al., 1995). After BSS processing, the data were segmented into 2-s time windows, and segments with amplitudes exceeding ±80 μV were discarded. The processed data were then saved for further analysis (Pascual-Marqui et al., 1995).

#### EEG microstate analysis

Our experiments in this paper are carried out on the platform of MATLAB (R2023a) and the corresponding toolbox of EEGLAB (2023.0), with the following specific steps. (1) The initial 2 min of both the resting state and the post-acupuncture state was deleted, and the reference channel was converted to an average reference. (2) Bandpass filtering in the 2–20 Hz range was performed using a finite pulse filter to reduce artifacts. (3) The global field power (GFP) was calculated. The GFP was calculated to describe the overall change in the EEG signal of all electrodes at a given time. The EEG data at the local maximum were selected as discrete EEG microstates (Equation 1). (4) Cluster analysis at the individual level was performed. The data before and after acupuncture were analyzed by k-means clustering separately, and the clustering maps of each subject were averaged to obtain the clustering topography of each subject under different conditions. The optimal number of clusters was determined using cross-validation criteria, with the number of microstate categories set to 4 based on previous research (Koenig et al., 2002). The maximum difference in topographies during clustering was explained by global explained variance (GEV) (Ricci et al., 2020). (5) The microstate parameters were reported. Microstate parameters, including duration, occurrence, contribution, and TP, are used to characterize the changes in EEG microstates before and after acupuncture. Duration refers to the average length of time that each microstate remains stable when it appears, and occurrence refers to the average number of occurrences per second during the appearance and stability of a microstate. Contribution represents the proportion of each microstate in the total time of the record. TP represents the probability of transition from one microstate to another (Ricci et al., 2022).


(1) $$GFP\left(t\right)=\sqrt{\frac{\Sigma_{i=1}^{N}v_{\dot{\nu }}{\left(t\right)}^{2}}{N}}$$

[*N* = number of electrodes, *Vi(t)* = measured voltage of electrode *i* at time *t*, and *i* = electrode *i*].

### Statistical analysis

All the data were analyzed using IBM SPSS 26.0 software. The UDVA, CS values, and microstate parameters were compared before and after acupuncture by Wilcoxon rank sum test separately, and we further performed false discovery rate (FDR) correction on the CS values and microstate parameters (Gu et al., 2022). Additionally, Spearman’s correlation and linear regression analysis were conducted between visual function data and EEG microstate parameters, separately. *p* values <0.05 were considered statistically significant.

## Results

### UDVA result

**Figure 4:**
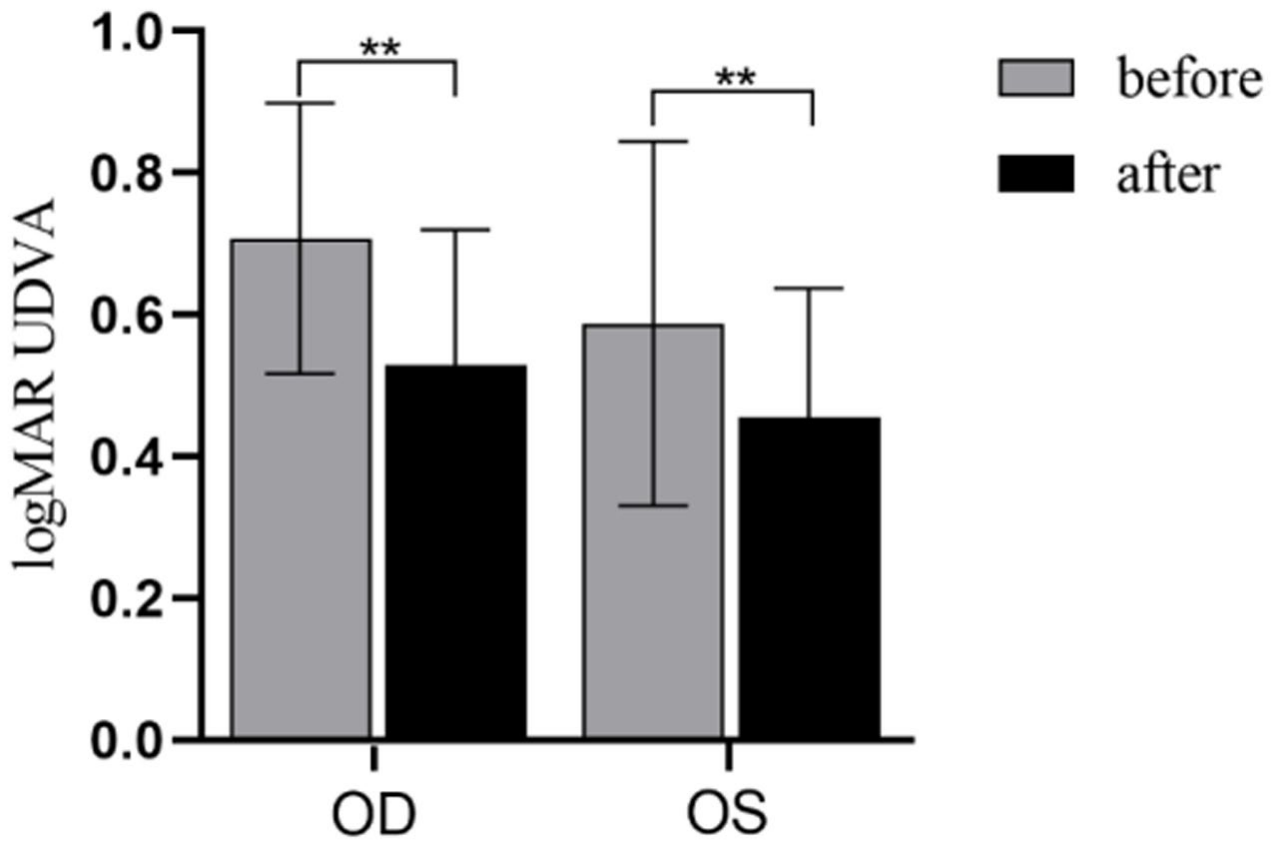
UDVA changes before and after acupuncture. OD, Right eye; OS, Left eye. **p < 0.01.

**Figure 5:**
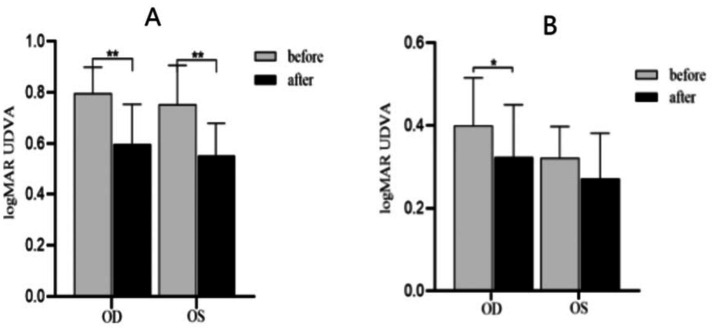
UDVA changes before and after acupuncture. OD, Right eye; OS, Left eye. **(A)** SE ≤ −0.50D and > −3.00D, **(B)** SE ≤ −3.00D and > −6.00D, **p < 0.01, *p < 0.05.

As shown in Figure 4, SE ≤ −0.50D and > −6.00D, compared with pre-acupuncture, the UDVA of both eyes increased significantly (*P*_OD_ = 0.001; *P*_OS_ = 0.004). To further evaluate the improvement effect of acupuncture on UVDA among different levels of myopia, we conducted a stratified analysis based on different spherical equivalent levels (Figure 5). SE ≤ −0.50D and > −3.00D, there was a significant improvement in UDVA of both eyes after acupuncture at GB37 (*P*_OD_ = 0.003; *P*_OS_ = 0.005); SE ≤ −3.00D and > −6.00D, UDVA in the right eye improved significantly (*P*_OD_ = 0.017; *P*_OS_ = 0.092).

### CS result

**Figure 6:**
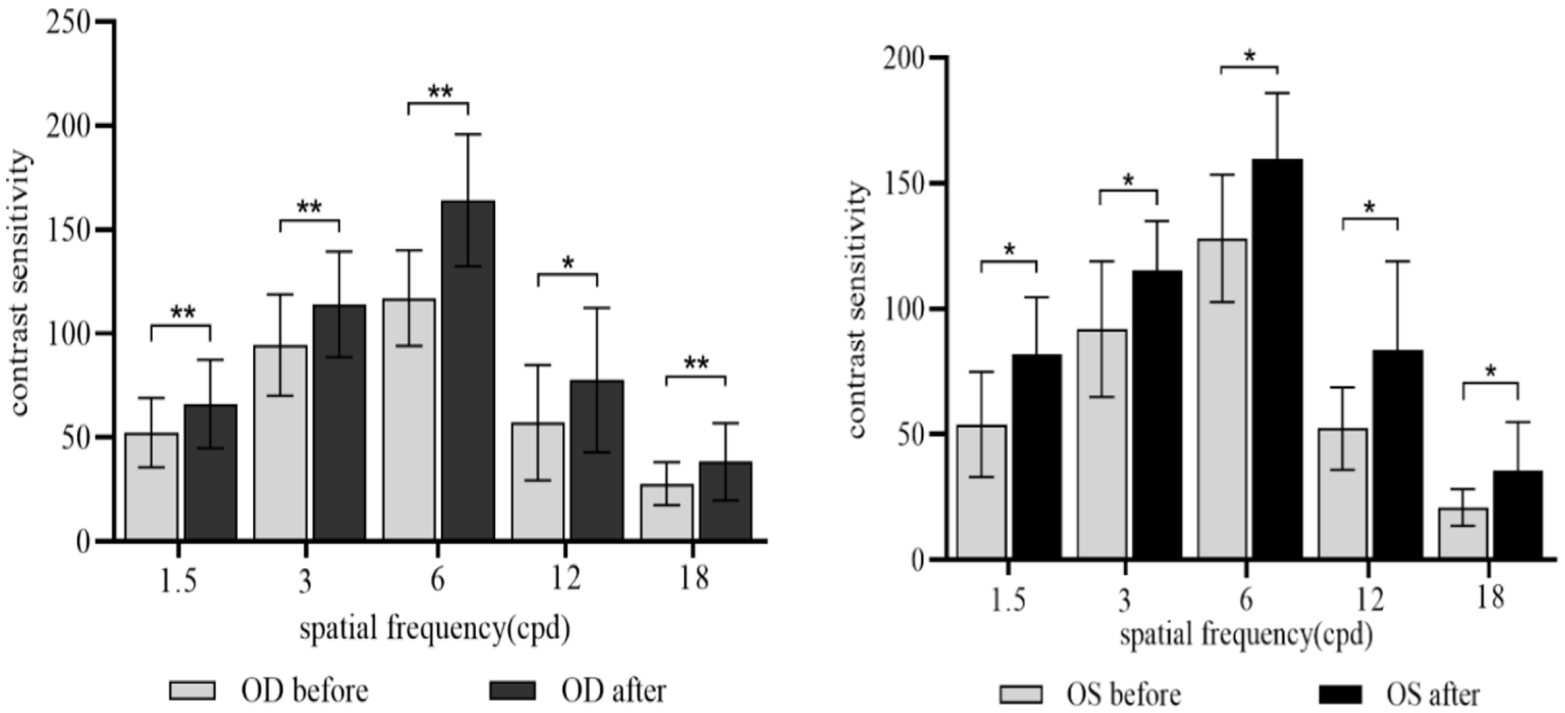
CS for each spatial frequency before and after acupuncture. OD, Right eye; OS, Left eye. *p_(FDR)_ < 0.05, **p_(FDR)_ < 0.01.

**Figure 7:**
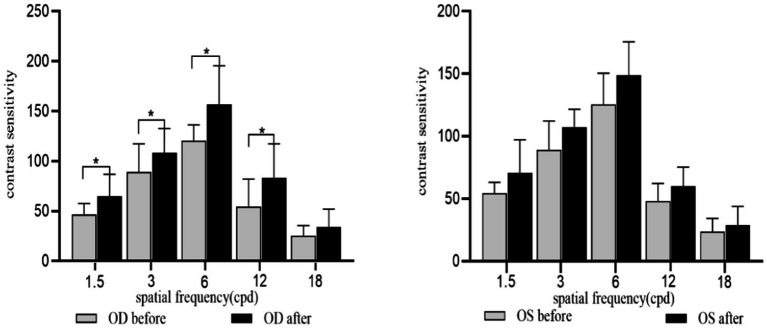
CS for each spatial frequency before and after acupuncture for SE ≤ −0.50D and > −3.00D. OD, Right eye; OS, Left eye. *p_(FDR)_ < 0.05, **p_(FDR)_ < 0.01.

**Figure 8:**
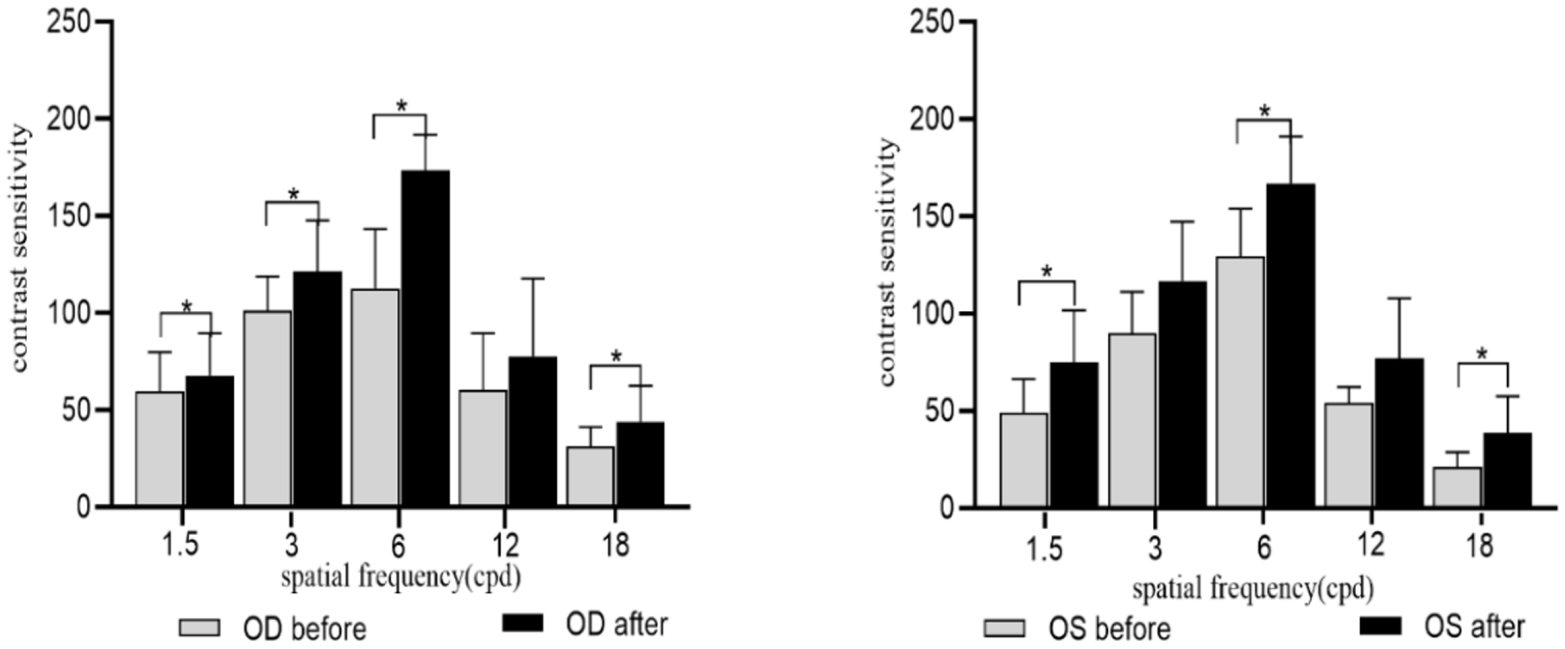
CS for each spatial frequency before and after acupuncture for SE ≤ −3.00D and > −6.00D. OD, right eye; OS, Left eye. *p_(FDR)_ < 0.05, **p_(FDR)_ < 0.01.

As shown in Figure 6, SE ≤ −0.50D and > −6.00D, the CS value increased at each spatial frequency of both eyes after acupuncture at GB37 [for OD: *P*_1.5cpd(FDR)_ = 0.004, *P*_3cpd(FDR)_ = 0.004, *P*_6cpd(FDR)_ = 0.004, *P*_12cpd(FDR)_ = 0.015, and *P*_18cpd(FDR)_ = 0.004; for OS: *P*_1.5cpd(FDR)_ = 0.015, *P*_3cpd(FDR)_ = 0.022, *P*_6cpd(FDR)_ = 0.022, *P*_12cpd(FDR)_ = 0.023, and *P*_18cpd(FDR)_ = 0.041]. At the same time, we conducted a stratified analysis among different levels of myopia to assess the effect of acupuncture on CS at different spatial frequencies. SE ≤ −0.50D and > −3.00D, the CS in the right eye showed significant improvement at spatial frequencies of 1.5, 3, 6, and 12 after acupuncture at GB37 [*P*_1.5cpd(FDR)_ = 0.020, *P*_3cpd(FDR)_ = 0.024, *P*_6cpd(FDR)_ = 0.024, *P*_12cpd(FDR)_ = 0.024], while there were no significant differences in the left eye. SE ≤ −3.00D and > −6.00D, there was a significant improvement in CS in both eyes at spatial frequencies of 1.5, 6, and 18 [for OD: *P*_1.5cpd(FDR)_ = 0.027, *P*_6cpd(FDR)_ = 0.034, *P*_18cpd(FDR)_ = 0.027; for OS: *P*_1.5 cpd(FDR)_ = 0.026, *P*_6cpd(FDR)_ = 0.020, *P*_18cpd(FDR)_ = 0.028] (Figures 7, 8).

### EEG microstate result

#### Global explained variance

**Figure 9:**
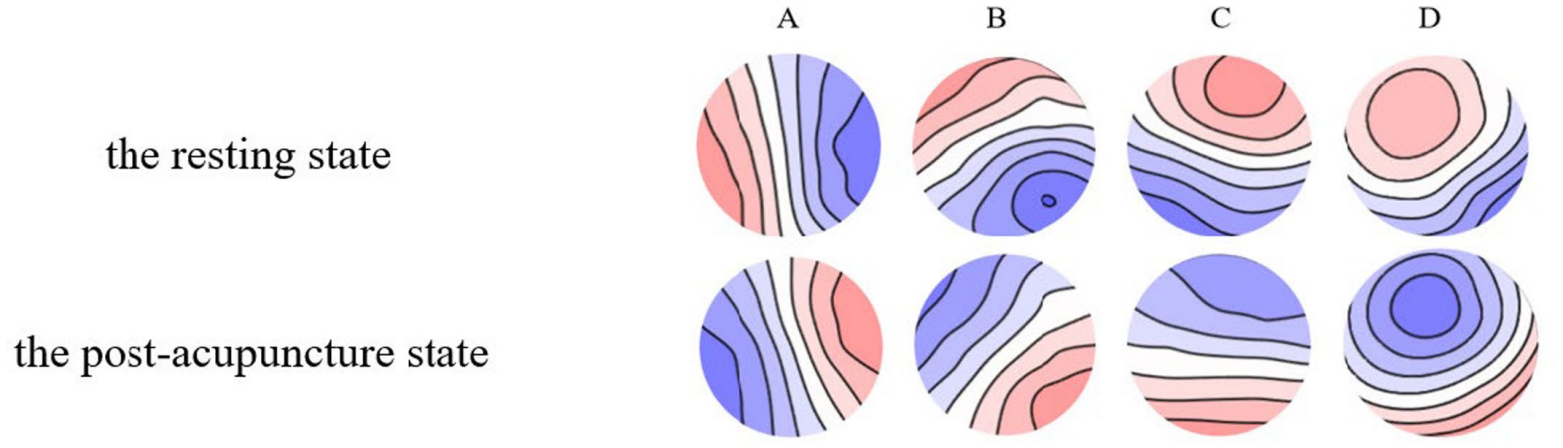
**(A–D)** EEG microstate maps during the resting state and the post-acupuncture state.

Figure 9 illustrates the topography of four EEG microstate types during the resting state and post-acupuncture state at GB37 point. The global explained variance (GEV) of the resting state and the post-acupuncture state were 79.84 and 77.30%, respectively. There was no significant difference between them.

#### Microstate parameters

**Figure 10:**
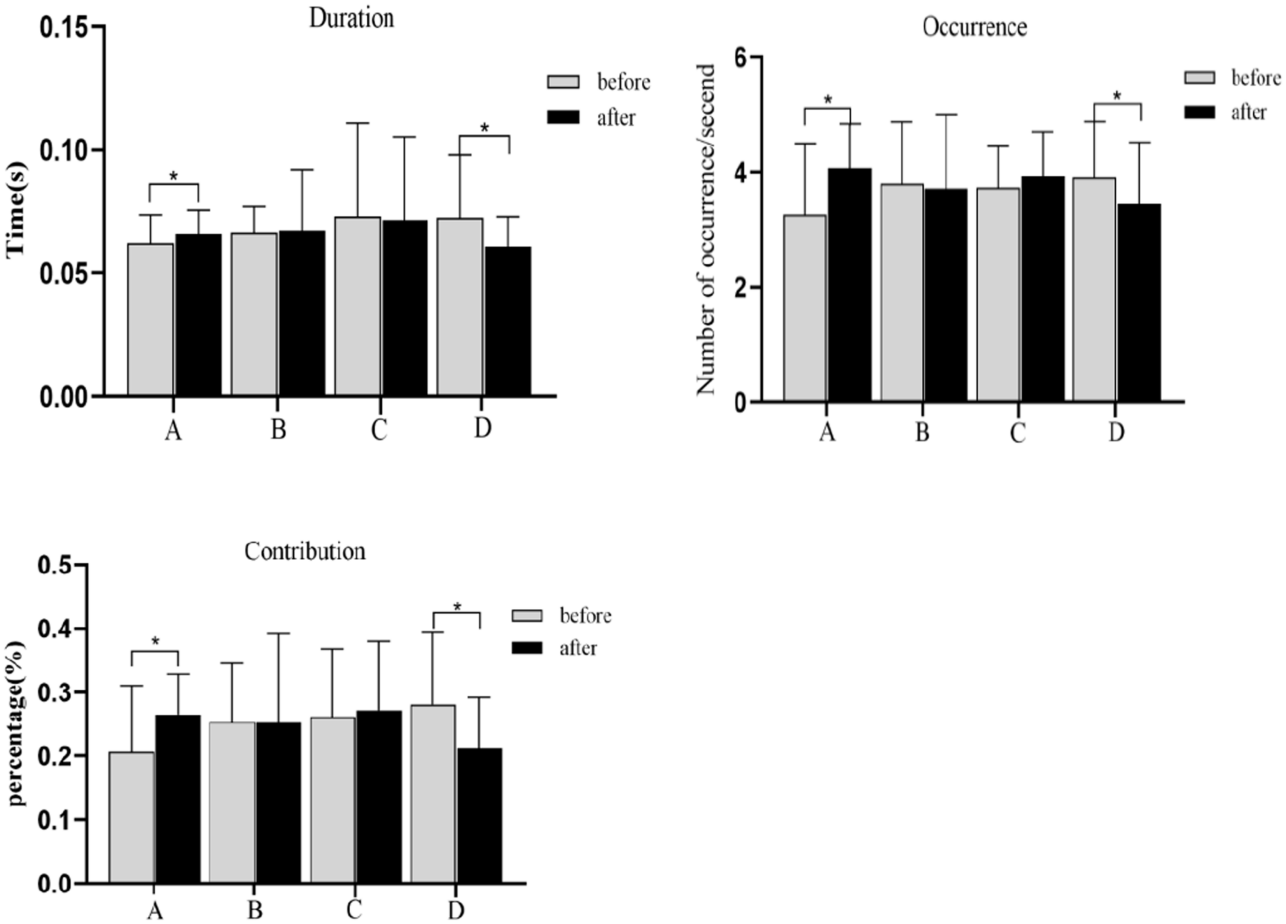
Duration, occurrence, contribution changes of microstate A and D during the resting state and the post-acupuncture state. *p_(FDR)_ < 0.05. Compared with the resting state, the TP between microstates A, B, C, and D have no significant difference during the post-acupuncture state (Figure 8).

Compared with the resting state, the duration, occurrence, and contribution of microstate A were significantly increased after acupuncture [*P*_duration A(FDR)_ = 0.034, *P*_occurrence A(FDR)_ = 0.016, *P*_contribution A(FDR)_ = 0.014], while those of microstate D were decreased significantly [*P*_duration D(FDR)_ = 0.034, *P*_occurrence D(FDR)_ = 0.033, *P*_contribution D(FDR)_ = 0.014]. The duration, occurrence, and contribution of microstate B and microstate C had no statistical significance between the resting state and the post-acupuncture state (Figure 10).

### Correlation between EEG microstates and visual function

#### Correlation between EEG microstates and UDVA

**Figure 11:**
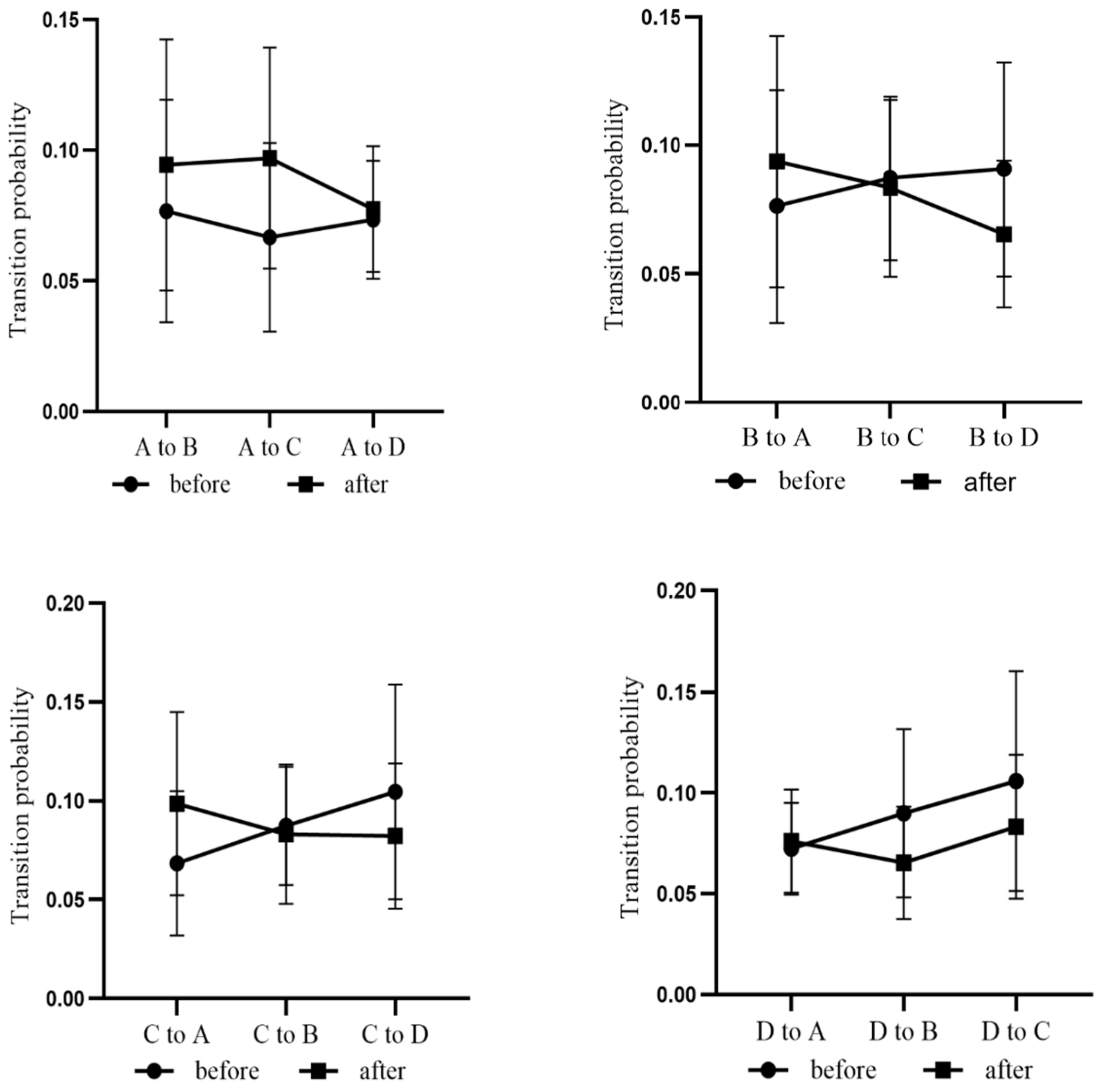
TP between different microstates during the resting state and the post-acupuncture state.

We performed a Spearman’s correlation analysis of the duration, occurrence and contribution between microstate A and microstate D and the monocular UDVA. And there was no correlation between them (Figure 11).

#### Correlation between EEG microstates and CS

**Figure 12:**
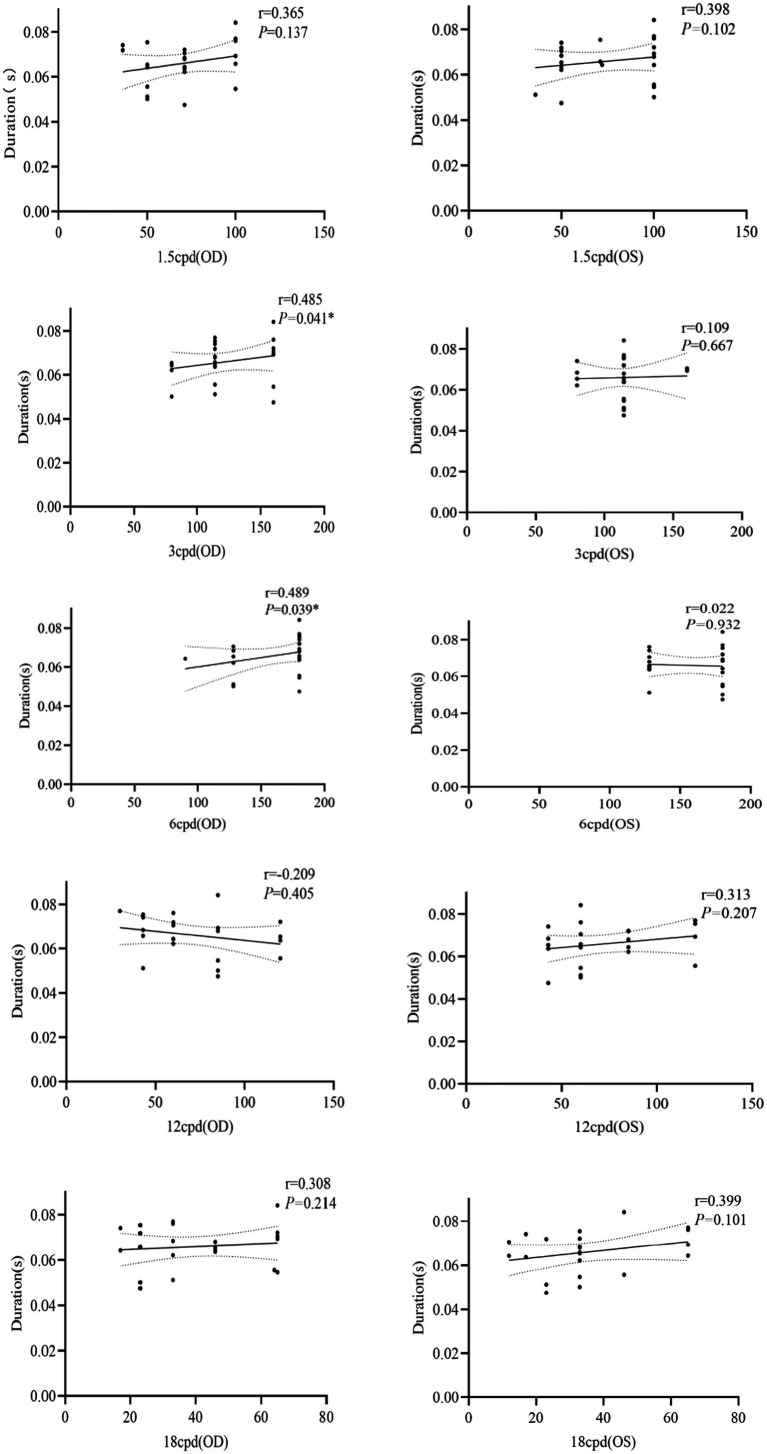
Correlation between duration of microstate A and CS at different spatial frequencies. OD for the right eye, OS for the left eye, *p < 0.05.

We also performed a Spearman’s correlation analysis of the duration, occurrence, and contribution between microstate A and microstate D and the monocular CS. As shown in Figure 12, the duration of microstate A was positively correlated with CS of 3 and 6 cpd for the oculus dexter (*r* = 0.485, *p* = 0.041; *r* = 0.489, *p* = 0.039). The duration of microstate A and CS of left space frequencies and had no correlation. The remaining parameters of microstate A and microstate D and CS had also no correlation.

## Discussion

This study aims to investigate the impact of GB37 acupuncture on visual function and electroencephalographic activity in myopic patients to explore the correlation between improved visual function and changes in brain activity. Our findings reveal that acupuncture at GB37 can improve UDVA and CS at all spatial frequencies in myopic patients. Acupuncture can increase the duration, occurrence, and contribution of microstate A, while those of microstate D were decreased. In addition, the duration of microstate A was positively correlated with the CS. However, there was no correlation between UDVA and EEG microstates.

Electroencephalography microstates are fundamental units of information processing that reflect dynamic changes in brain network activity. Based on fMRI-EEG multimodal studies, neural components generating microstates coincide with resting state networks identified independently through fMRI, and that EEG microstates show a strong correlation with these fMRI resting state networks (Danna et al., 2020). Microstate A exhibits a strong correlation with activity in the left insula, occipital cortex, and anterior cingulate cortex, influenced by visual stimuli and related to visual processing. According to the previous research, microstate A is closely related to the activation of visual network (Milz et al., 2016; Tarailis et al., 2024). And visual network is involved in self-visualization, autobiographical memory, and scene visualization, which is believed to be spatially related to the bilateral occipital regions (Custo et al., 2017). It plays a fundamental and stimulus-related role in emotion cognition (Rolls, 2019). Compared to insomnia patients, normal individuals have enhanced visual analysis skills and processing abilities (Giora et al., 2017). Milz et al. (2016) found during visual task, the duration, occurrence, and coverage of microstate A were increasing. Consistent with this research, our study found that the duration of microstate A was significantly increased after acupuncture at GB37 (Figure 10). Visual stimuli triggered widespread activation across the temporal and occipital cortices, alongside partial activation in the parietal and prefrontal cortex (Cléry et al., 2020). Visual networks become more active during the execution of a visual attention task (Li et al., 2019). This suggested an enhanced role of the visual network within the brain’s functional networks after acupuncture, which benefits the processing of visual information. Furthermore, the occurrence and contribution of microstate A were also increased (Figure 10), which indicated that the visual network was continuously activated following acupuncture, thereby stimulating the brain’s extraction of visual information.

Microstate D is closely related to the dorsal attention network (DAN), reflecting cognitive selection of sensory stimuli and responses (Kim, 2014). The EEG-fMRI synchronization study showed that microstate D was associated with the right frontal and parietal cortex, which was belonged to the attention network (Britz et al., 2010) and mainly reflected attention, focus shifting and repositioning (Li W. et al., 2023). DAN primarily oversees top-down attentional processes (Zhan et al., 2016), and its activation decreases during visual tasks that do not require external attention (Vossel et al., 2014; Eryurek et al., 2022). The decrease of microstate D parameters is considered to be related to that the DAN is inhibited during the acupuncture manipulation (Si et al., 2021), while the increase of microstate indexes may be the function activity of the neural network (Zappasodi et al., 2017). It has been shown that, compared to individuals with insomnia (Wei et al., 2018), vertigo (Li Y. N. et al., 2022), and attention deficit states (Luo et al., 2021), normal individuals show significantly reduced in duration, occurrence, and contribution of microstate D. Our present study found these metrics of microstate D were significantly decreased after acupuncture at GB37 (Figure 10), consistent with previous researches. We further speculate that acupuncture could inhibit the information processing of DAN with the auditory and visual sensory network, which could be evidenced by microstate D’s decrease, and help reduce the subject’s top-down attention to perceive sensory stimuli (Si et al., 2021). A study indicated that acupuncture at the GB37 point could affect the neuronal activity in the visual pathway (Danna et al., 2020). Our results suggest that acupuncture at GB37 can suppresses the activity of the dorsal attention network. We hypothesize that this change is related to the suppression of information processing in attentional networks associated with auditory and verbal networks, thereby enhancing the extraction of visually relevant information. However this change is not related to the alterations in of visual function such as UDVA and CS, and its implications warrant further exploration.

Previous simultaneous fMRI-EEG research demonstrated that EEG microstates can reveal the dynamic patterns of EEG activities (Michel and Koenig, 2018), with transition probabilities particularly speculating the dynamic switching patterns of underlying functional networks (Britz et al., 2010). However, there are no differences in the transition probability between different microstates (Figure 11). Preliminary study by our research group have shown that acupuncture at *Taiyang* acupuncture can reduce the contribution of microstate C and the transition probability between microstate A and C (Su et al., 2023), which is inconsistent with the results of our present studies. It may be due to the distinct neural regulatory pathways that affect brain activity following acupuncture at different acupoints. Different acupuncture points can target distinct brain networks, leading to similar clinical outcomes (Dong et al., 2022). In future studies, it is necessary to increase the sample size for further investigation.

Furthermore, we analyzed the correlation between EEG microstates and UDVA and CS. The results showed that the duration of microstate A was positively correlated with CS (Figure 8), which indicated that acupuncture at GB37 may improve CS by activating the visual network and increasing the steady state of microstate A. The results of this study indicate that the duration parameter of microstate A can serve as an effective indicator for the improvement of visual function following acupuncture at GB37 point.

### Limitations

This study has a few limitations. First, recruiting more participants would strengthen the evidence for further investigating the correlation between visual function and neural responses in future research. Second, This study focuses solely on the acupuncture point *Guangming* as a basic unit to explore the neural regulatory effects of acupuncture on visual function, which did not include placebo points. Future research should further explore the neural regulation mechanisms related to EEG microstates and other acupuncture points, as well as placebo points. Third, in this study, a control group with emmetropia was not included to confirm whether the effect of acupuncture is specific to patients with myopia. In future research, we will incorporate these patients for further validation. Forth, we did not evaluate the long-term effects of acupuncture on visual function and brain network status. Future studies should include follow-up assessments at different time points to evaluate the longevity of acupuncture’s impact.

## Conclusion

In summary, acupuncture at GB37 can improve the UDVA and CS in myopic patients. The duration, occurrence frequency and contribution of microstate A and microstate D underwent significant changes. However, there are no differences in the transition probability between the microstates A, B, C, and D, which means that acupuncture has no discernible impact on the overall neural activity patterns of the brain. Furthermore, the duration of microstate A was positively correlated with CS, with no significant correlation for microstates B–D. This suggests that acupuncture at GB37 can improve the visual function of myopic patients, which is closely related to microstate A.

## Data Availability

The original contributions presented in the study are included in the article/supplementary material, further inquiries can be directed to the corresponding authors.
